# Symposium on Phlegmons of the Upper Respiratory Tract--Report of a Case

**Published:** 1913-09

**Authors:** F. E. Hopkins

**Affiliations:** Springfield


### Symposium on Phlegmons of the Upper Respiratory Tract--Report of a Case.

This case illustrates the possibility of erosion of a large blood-vessel. The patient was a male 26 years of age, of poor resistance because of leading an irregular life and having had a recent acute illness. He had suffered from measles and while convalescing took cold. There was marked swelling on the left side with severe pain, but the patient was not prostrated and at no time did his temperature go above 101 degrees F., or his pulse above 80 or 90. Deep incisions were made, but these yielded no pus, and there was no evidence of pointing. Two days later a hemorrhage occurred which was controlled by pressure. On the following day a terrific hemorrhage occurred and quickly proved fatal. Such an examination as could be made immediately following death showed rupture through the posterior pillar, the flood from the eroded carotid finding exit there. Such a mass of cellular infiltration should be explored with a blunt instrument, even the finger, following unfruitful incision. The wonder is not that phlegmons threatening life occasionally develop, but rather, considering the frequency with which infections of this region occur, that they are so rare that an active professional life may pass and not a single one come under observation. Microscopic findings are of little value in determining the treatment of these cases. Early and effective drainage is the best assurance of a favorable prognosis. Suffocation from flooding of the larynx by the sudden rupture of an abscess has been reported, and tracheotomy has been required because of closure of the pharynx by infiltration and edema, but the complication I wish to emphasize is that of erosion of blood vessels by the necrotic process.—Dr. F. E. Hopkins, Springfield.

